# Antimicrobial Resistance and CRISPR Typing Among *Salmonella* Isolates From Poultry Farms in China

**DOI:** 10.3389/fmicb.2021.730046

**Published:** 2021-09-16

**Authors:** Cui Li, Yulong Wang, Yufeng Gao, Chao Li, Boheng Ma, Hongning Wang

**Affiliations:** Animal Disease Prevention and Food Safety Key Laboratory of Sichuan Province, Key Laboratory of Bio-Resource and Eco-Environment of Ministry of Education, College of Life Sciences, Sichuan University, Chengdu, China

**Keywords:** *Salmonella*, CRISPR-Cas, antimicrobial resistance, molecular typing, CRISPR array

## Abstract

Although knowledge of the clustered regularly interspaced short palindromic repeat (CRISPR)-Cas system has been applied in many research areas, comprehensive studies of this system in *Salmonella*, particularly in analysis of antibiotic resistance, have not been reported. In this work, 75 *Salmonella* isolates obtained from broilers or broilers products were characterized to determine their antimicrobial susceptibilities, antibiotic resistance gene profiles, and CRISPR array diversities, and genotyping was explored. In total, 80.00% (60/75) of the strains were multidrug resistant, and the main pattern observed in the isolates was CN-AZM-AMP-AMC-CAZ-CIP-ATM-TE-SXT-FOS-C. The resistance genes of streptomycin (*aadA*), phenicol (*floR-like and catB3-like*), β-lactams (*bla*_TEM_, *bla*_OXA_, and *bla*_CTX_), tetracycline [*tet(A)-like*], and sulfonamides (*sul1* and *sul2*) appeared at higher frequencies among the corresponding resistant isolates. Subsequently, we analyzed the CRISPR arrays and found 517 unique spacer sequences and 31 unique direct repeat sequences. Based on the CRISPR spacer sequences, we developed a novel typing method, CRISPR locus three spacer sequences typing (CLTSST), to help identify sources of *Salmonella* outbreaks especially correlated with epidemiological data. Compared with multi-locus sequence typing (MLST), conventional CRISPR typing (CCT), and CRISPR locus spacer pair typing (CLSPT), discrimination using CLTSST was weaker than that using CCT but stronger than that using MLST and CLSPT. In addition, we also found that there were no close correlations between CRISPR loci and antibiotics but had close correlations between CRISPR loci and antibiotic resistance genes in *Salmonella* isolates.

## Introduction

*Salmonella* is one of the main causes of bacterial gastroenteritis worldwide. To date, more than 2,600 *Salmonella* serotypes have been reported globally (Graziani et al., 2017). Moreover, approximately one-tenth of individuals worldwide are infected by non-typhoidal *Salmonella enterica* isolates annually resulting in 33 million deaths [*Salmonella* (non-typhoidal), 2018]. However, among *Salmonella* serotypes, only a limited number causes human infection (Uzzau et al., 2000), such as *Salmonella enterica* serotype Enteritidis (*S. Enteritidis*) and *Salmonella enterica* serotype Typhimurium (*S. Typhimurium*); identification of these infective serotypes is important for public health. *Salmonella* is widespread in domestic and wild animals, particularly poultry and poultry products which have been identified as important sources of human salmonellosis (Sanchez et al., 2002; Antunes et al., 2016; McWhorter and Chousalkar, 2019). For example, in 2018, approximately 207 million eggs from Rose Acre Farms (Seymour, Indiana, United States) were contaminated with *Salmonella enterica* serotype Braenderup, infecting 45 people, and were recalled across the United States (Centers for Disease Control and Prevention, 2018a). Similarly, eggs from Gravel Ridge Farms (Cullman, Alabama, United States) contaminated with *S. Enteritidis*, infected 44 people and had to be recalled (Centers for Disease Control and Prevention, 2018b), and a raw frozen chicken product contaminated with multidrug-resistant *Salmonella enterica* serotype Infantis (*S. Infantis*) infected 129 people (Centers for Disease Control and Prevention, 2019). Currently, the most commonly used treatment method for *Salmonella* infection is antibiotic therapy. However, the unnecessary long-term use of antibiotics has led to the development of *Salmonella* resistance, which poses a major challenge to the treatment of salmonellosis. In particular, there are many serotypes of *Salmonella*, how to quickly type the strain and trace the source of salmonellosis outbreaks remains to be resolved.

Different molecular typing techniques, such as pulse-field gel electrophoresis (PFGE), multi-locus sequence typing (MLST), and multi-locus variable number tandem repeat (VNTR) analysis, have been developed to track the origins of bacterial disease. PFGE typing has a high resolution for analyzing the homology of pathogenic bacteria from different sources, tracing the origin of foodborne disease outbreaks, determining the diversity of pathogenic bacteria in food, and evaluating the relationships between pathogenic bacteria in food and the related environment (Pang et al., 2007; Best et al., 2009). However, there are no uniform standards for naming, making it difficult to share data and to distinguish among the long-term evolution process from small changes between different strains of the same clone. Although MLST technology compensates for the deficiency of PFGE, it has some limitations for the application of typing in some highly homologous pathogens, due to the conservation of housekeeping genes (Turki et al., 2014; Campioni et al., 2015).

In recent years, innovative and powerful typing methods based on clustered regularly interspaced short palindromic repeat (CRISPR) loci have been developed (Barrangou and Dudley, 2016) and applied to the typing of a variety of bacteria, such as *Salmonella* (Fabre et al., 2012) and *Escherichia coli* (Long et al., 2020). Through the study of *S. Heidelberg* typing, it is found that CRISPR typing can be useful for *Salmonella* foodborne outbreaks subtyping as well as serve as a complimentary tool to determine source attribution in foodborne outbreaks (Vincent et al., 2018; Yousfi et al., 2020). Researchers combined the multi-virulence gene locus sequence typing (MVLST) scheme with CRISPR typing to develop a CRISPR-MVLST method, which was used to genotype 171 *Salmonella* strains from nine serotypes. The results of this method were better than CRISPR and MLST and could be used as an important genotyping method in *Salmonella* outbreaks (Liu et al., 2011). Upon comparing the effects of PFGE and CRISPR-MVLST on typing 84 strains of *Salmonella enterica* serotype Newport, researchers found that the Simpson index D values of the two methods were both higher than 0.95, suggesting complementary with each other in practice (Shariat et al., 2013b). A new typing method, CRISPR locus spacer pair typing (CLSPT), analyzes only the two newly incorporated spacers adjoining the leader sequence in the two CRISPR loci. Use of this method to analyze 82 *Salmonella* strains of 21 serotypes showed that although this method was weaker than conventional CRISPR typing (CCT) and PFGE typing, it was better than MLST and did not require analysis of the full CRISPR sequence map; it was also low cost and more practical, requiring only simple operation procedures (Li et al., 2014). Sequencing of 156 *Salmonella* strains showed that the evolutionary pattern reflected by the CRISPR locus was different from that of phylogeny, and horizontal gene transfer and changes in the shared environment could affect the phylogenetic distribution of *Salmonella* (Timme et al., 2013). Among these above methods, it is either cumbersome or ignoring the differences in spacer sequences. Thus in order to make up for this shortcoming, a novel method should be developed.

Since the discovery of the CRISPR locus in the 1980s, the functions and applications of CRISPR in prokaryotes have attracted much attention. For example, its spacer sequence has been used as an auxiliary tool for a variety of bacterial typing studies, epidemiological investigations, and evolutionary analyses. The CRISPR array has a high degree of polymorphism, which includes the unique biological and geographical characteristics of bacteria. However, few studies have reported its relationship with bacterial drug resistance, and the results of these studies in different bacteria have been inconsistent. For example, in *Klebsiella pneumonia*, *Neisseria meningitidis*, and *Vibrio cholera*, partly antibiotic resistance genes (ARGs) were positively associated with CRISPR-Cas system (Shehreen et al., 2019). But it is interesting that on the time scale of evolution, CRISPR-Cas has no significant effect on horizontal gene transfers (Gophna et al., 2015). It also has proved that the CRISPR-Cas system in *E. coli* does not affect the spread of plasmid and antibiotic (Touchon et al., 2012). And *Shigella* strains could regulate the activity of the CRISPR-Cas system by the insertion sequence (IS) elements that were identified in cas genes and then acquired exogenous resistance genes (Chen et al., 2019). In *Salmonella*, genome analysis has suggested that generally there are two CRISPR loci and a single type of CRISPR-Cas system, type I (mainly I–E) (Jansen et al., 2002; Grissa et al., 2007). Therefore, in this study, we aimed to analyze the occurrence and diversity of CRISPR arrays using whole-genome sequencing (WGS) data from 75 *Salmonella* isolates. Additionally, we established a new typing method based on CRISPR for *Salmonella*, and we further explored the relationships between the CRISPR-Cas system and drug resistance in *Salmonella*.

## Materials and Methods

### *Salmonella* Strains and Antimicrobial Susceptibility Testing

In total, 75 *Salmonella* isolates were collected from different broilers or broilers products in eight poultry farms at three provinces (Hebei, Sichuan, and Shandong) and Chongqing municipality of China between 2018 and 2019. *Salmonella* isolation was performed as previously reported (Cai et al., 2016). In brief, first, the pre-enrichment step of *Salmonella* was carried out by culturing the samples in 10mL buffered peptone water (BPW, Beijing Land Bridge Technology Co., Ltd., China) at 37 °C with 180rpm for 16h. Then, 100μL of the BPW was cultured in 10mL Rappaport’s Broth (MM, Bejing Land Bridge Technology Co., Ltd., China) at 42 °C with 180rpm for 24h. Subsequently, the MM culture was streaked into xylose lysine tergitol 4 (XLT4, Bejing Land Bridge Technology Co., Ltd., China) at 37 °C for 36h in a microbial incubator.

Immunological serotyping of *Salmonella* isolates was confirmed by slide agglutination for flagellar (H) and somatic (O) antigens using commercially available antisera (Tianrun Bio-Pharmaceutical, Ningbo, China), according to the manufacturer’s instructions. The antibiotic susceptibility of these isolates was tested using the disk diffusion method on Mueller-Hinton agar (MHA) according to the guidelines provided by the Clinical and Laboratory Standards Institute (CLSI) (Clinical and Laboratory Standards Institute, 2019), including gentamycin (CN, 10μg), azithromycin (AZM, 15μg), ampicillin (AMP, 10μg), amoxicillin/clavulanic acid (AMC, 20/10μg), ampicillin/sulbactam (SAM, 10/10μg), cefoxitin (FOX, 30μg), ceftazidime (CAZ, 30μg), polymyxin B (PB, 300IU), meropenem (MEM, 10μg), ceftazidime (CIP, 5μg), aztreonam (ATM, 30μg), tetracycline (TE, 30μg), trimethoprim/sulfamethoxazole (SXT, 1.25/23.75μg), chloramphenicol (C, 30μg), fosfomycin (FOS, 200μg), and nalidixic acid (NA, 30μg). *Escherichia coli* strain ATCC 25922 was used as a quality control organism in all antimicrobial susceptibility tests.

Immunological serotyping of *Salmonella* isolates was confirmed by slide agglutination for flagellar (H) and somatic (O) antigens using commercially available antisera (Tianrun Bio-Pharmaceutical, Ningbo, China), according to the manufacturer’s instructions. The antibiotic susceptibility of these isolates was tested using the disk diffusion method on Mueller-Hinton agar (MHA) according to the guidelines provided by the Clinical and Laboratory Standards Institute (CLSI) Clinical and Laboratory Standards Institute, 2019, including gentamycin (CN, 10μg), azithromycin (AZM, 15μg), ampicillin (AMP, 10μg), amoxicillin/clavulanic acid (AMC, 20/10μg), ampicillin/sulbactam (SAM, 10/10μg), cefoxitin (FOX, 30μg), ceftazidime (CAZ, 30μg), polymyxin B (PB, 300IU), meropenem (MEM, 10μg), ceftazidime (CIP, 5μg), aztreonam (ATM, 30μg), tetracycline (TE, 30μg), trimethoprim/sulfamethoxazole (SXT, 1.25/23.75μg), chloramphenicol (C, 30μg), fosfomycin (FOS, 200μg), and nalidixic acid (NA, 30μg). *Escherichia coli* strain ATCC 25922 was used as a quality control organism in all antimicrobial susceptibility tests.

### WGS and Analysis of Antibiotic Resistance Genes

Genomic DNA was extracted from *Salmonella* isolates using a TIANamp Bacteria DNA Kit (Tiangen, Beijing, China), and WGS was carried out by Novogene Co., Ltd., Beijing, China. The sequencing results were analyzed on an Illumina MiSeq platform using 150 base paired-end reads, according to the manufacturer’s instructions. The gene fragments were assembled using SPAdes 3.13.0 (Bankevich et al., 2012). Next, the 75 *Salmonella* genome sequences were submitted to the Center for Genomic Epidemiology (CGE)^1^ and analyzed for ARGs.

### Analysis of CRISPR Arrays and Cas Genes

All CRISPR sequences were analyzed with CRISPRCasFinder^2^, which could be used to identify CRISPR arrays, cas genes, and the CRISPR-Cas type and subtype (Couvin et al., 2018). The results were visualized using CRISPRviz (Nethery and Barrangou, 2019). Weblogo^3^ was used for the analysis of the conservation of leader sequences and direct repeat (DR) sequences (Crooks et al., 2004). Direct repeat sequences were classified according to v1.3.0–2013 in the CRISPRmap (Lange et al., 2013). Secondary structure and minimum free energy (MFE) predictions were performed using the RNAfold web server^4^ (Mathews et al., 2004). CRISPRTarget^5^ was used to analyze spacer sequences (Biswas et al., 2013) and the cutoff score was 20. An unweighted pair group method with arithmetic mean (UPGMA) phylogenetic tree based on the CRISPR sequences was constructed to depict the clustering of subtypes determined by CRISPR diversity.

### Multi-Locus Sequence Typing (MLST)

The typing scheme utilized conserved sequences within the housekeeping genes (*aroC*, *dnaN*, *hemD*, *hisD*, *purE*, *sucA*, and *thrA*). The 75 *Salmonella* genome sequences were submitted to CGE, and MLST analyses were carried out on the CGE using MLST 2.0^6^. The minimum spanning tree was constructed using Phyloviz with the goeBURST Full MST algorithm based on the seven MLST loci. The discrimination index was evaluated based on Simpson’s index of diversity using a previously described equation (Hunter and Gaston, 1988).

### Conventional CRISPR Typing (CCT)

First, all spacer sequences in the two gene sequences of CRISPR1 and CRISPR2 were extracted, and the CRISPR1 and CRISPR2 spacer sequence profiles were then drawn and visualized using CRISPRviz.

### CRISPR Locus Spacer Pair Typing (CLSPT)

A spacer sequence in CRISPR1 and CRISPR2 was used, and this spacer sequence was the first spacer sequence close to the leader sequence. The first spacer sequence of the CRISPR1 leader sequence was combined with the first spacer sequence of the CRISPR2 leader sequence. These two spacer sequences were used as the total sequence for *Salmonella* strain typing.

### CRISPR Locus Three Spacer Sequences Typing (CLTSST)

During the evolution of the strain, the first captured exogenous nucleotide sequence displayed the original information for the strain, and the spacers of the same serotype had a certain degree of conservation. Therefore, this feature could be used to distinguish different serotype clusters. Thus, we developed a new method for *Salmonella* typing, called as CLTSST. Three spacer sequences that were the initial two spacer sequences (the furthest distance to the leader sequence) and latest spacer sequence close to the leader sequence were extracted, and these spacer sequences were then combined as the total sequence for strain typing.

## Results

### Serotyping and Antibiotic Susceptibility of *Salmonella*

Thirteen *Salmonella enterica* serotypes were identified from the 75 *Salmonella* isolates, including four strains of *S. Enteritidis*, 20 strains of *S. Indiana*, 11 strains of *S. Derby*, six strains of *S. Senftenberg*, three strains of *S. Kentucky*, 24 strains of *S. Typhimurium*, and other common epidemic serotypes (Supplementary Table S1).

**Figure 1:**
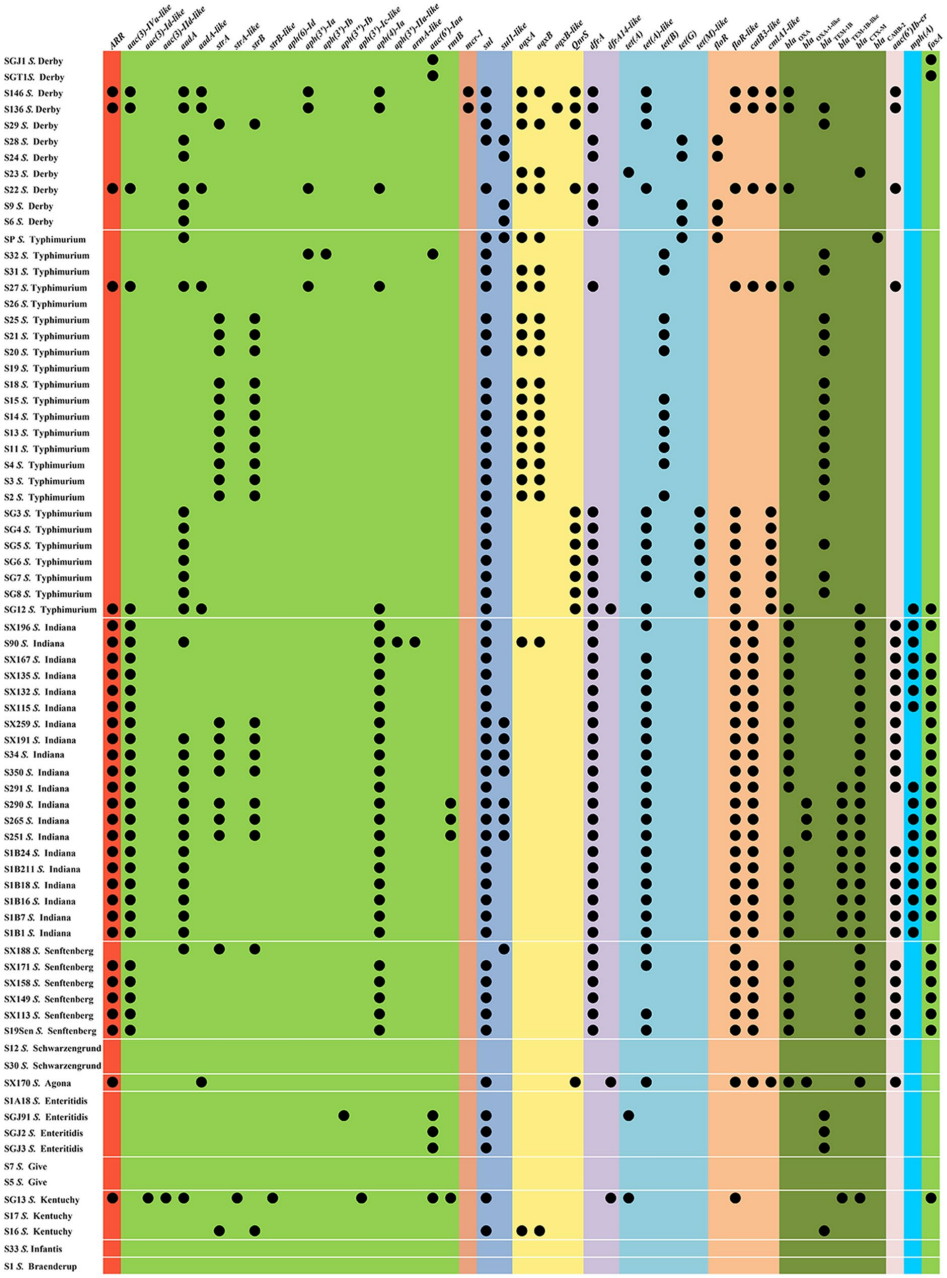
Resistance gene profiles from the *Salmonella* isolates.

The antimicrobial susceptibilities of 75 *Salmonella* isolates were observed (Table 1). More than two-thirds (69.33–80.00%) of the isolates were resistant to AMP, SXT, C, TE, and CIP, whereas almost half (40.00–42.67%) of the isolates were resistant to CN, AMC, FOS, and CAZ. Nearly one-third (36.00%) of the tested isolates exhibited resistance to ATM. Approximately one-quarter (22.67–25.33%) of the isolates was resistant to NA and AZM. The resistance rates to PB, MEM, SAM, and FOX were relatively low (0.00–2.67%). The drug resistance spectrum of the isolated bacteria (Table 1) showed that 80.00% (60/75) of the strains were multidrug-resistant bacteria that were resistant to three or more drug types, reflecting that antibiotic resistance in the isolated bacteria was prevalent with 25 different resistance patterns identified (Table 1). The main pattern in 14 of the isolates was CN-AZM-AMP-AMC-CAZ-CIP-ATM-TE-SXT-FOS-C. However, serotypes and resistance patterns were not correlated, and 47 resistance-associated genes were found in all isolates (Figure 1 and Supplementary Table S2). Among these resistance genes, *sul1*, *sul2*, *tet(A)-like*, *bla*_TEM_, *bla*_CTX_, *bla*_OXA_, *floR-like*, *catB3-like*, *aac(3)-Iva-like*, *aph(4)-Ia*, and *fosA* were found in *Salmonella* isolates at rates of 60.00, 62.67, 45.33, 44.00, 40.00, 41.33, 52.00, 40.00, 40.00, 40.00, and 37.33%, respectively.

**Table 1:** Antibiotic-resistant profiles of *Salmonella* isolates to different antibiotics.

Antibiotic-resistant profiles^a^	No. of the isolate(s)
CN-AZM-AMP-AMC-CAZ-CIP-ATM-TE-SXT-FOS-C	14
CN-AZM-AMP-AMC-CAZ-CIP-ATM-SXT-C-NA	1
CN-AZM-AMP-AMC-CAZ-ATM-TE-SXT-FOS-C	1
CN-AMP-AMC-CAZ-CIP-ATM-TE-SXT-FOS-C	8
AMP-AMC-CAZ-CIP-ATM-TE-SXT-FOS-C	1
CN-AMP-AMC-PB-CIP-TE-SXT-FOS-C-NA	2
CN-AMP-AMC-CAZ-CIP-ATM-SXT-FOS-C	1
CN-AZM-AMP-CAZ-CIP-TE-SXT-C	1
CN-AMP-AMC-CIP-TE-SXT-C-NA	1
CN-AMP-AMC-CIP-SXT-C-NA	1
AMP-AMC-CIP-TE-SXT-C	1
CN-AMP-CAZ-TE-SXT-C	1
CN-AMP-AMC-CIP-TE-C	1
AMP-CAZ-CIP-TE-C-NA	1
AMP-CIP-TE-SXT-C-NA	10
AMP-CAZ-ATM-FOS-C	1
AMP-CIP-SXT-C-NA	3
AMP-CIP-TE-SXT-C	3
CIP-TE-SXT-C	3
AMP-TE-C	1
TE-SXT-C	4
AMP-TE	1
FOS	2
AMP	2
none	10

a CN, gentamycin; AZM, azithromycin; AMP, ampicillin; AMC, amoxicillin/clavulanic acid; SAM, ampicillin/sulbactam; FOX, cefoxitin; CAZ, ceftazidime; PB, polymyxin B; MEM, meropenem; CIP, ciprofloxacin; ATM, aztreonam; TE, tetracycline; SXT, trimethoprim/sulfamethoxazole; C, chloramphenicol; FOS, fosfomycin, and NA, nalidixic acid.

### MLST Analysis

**Figure 2:**
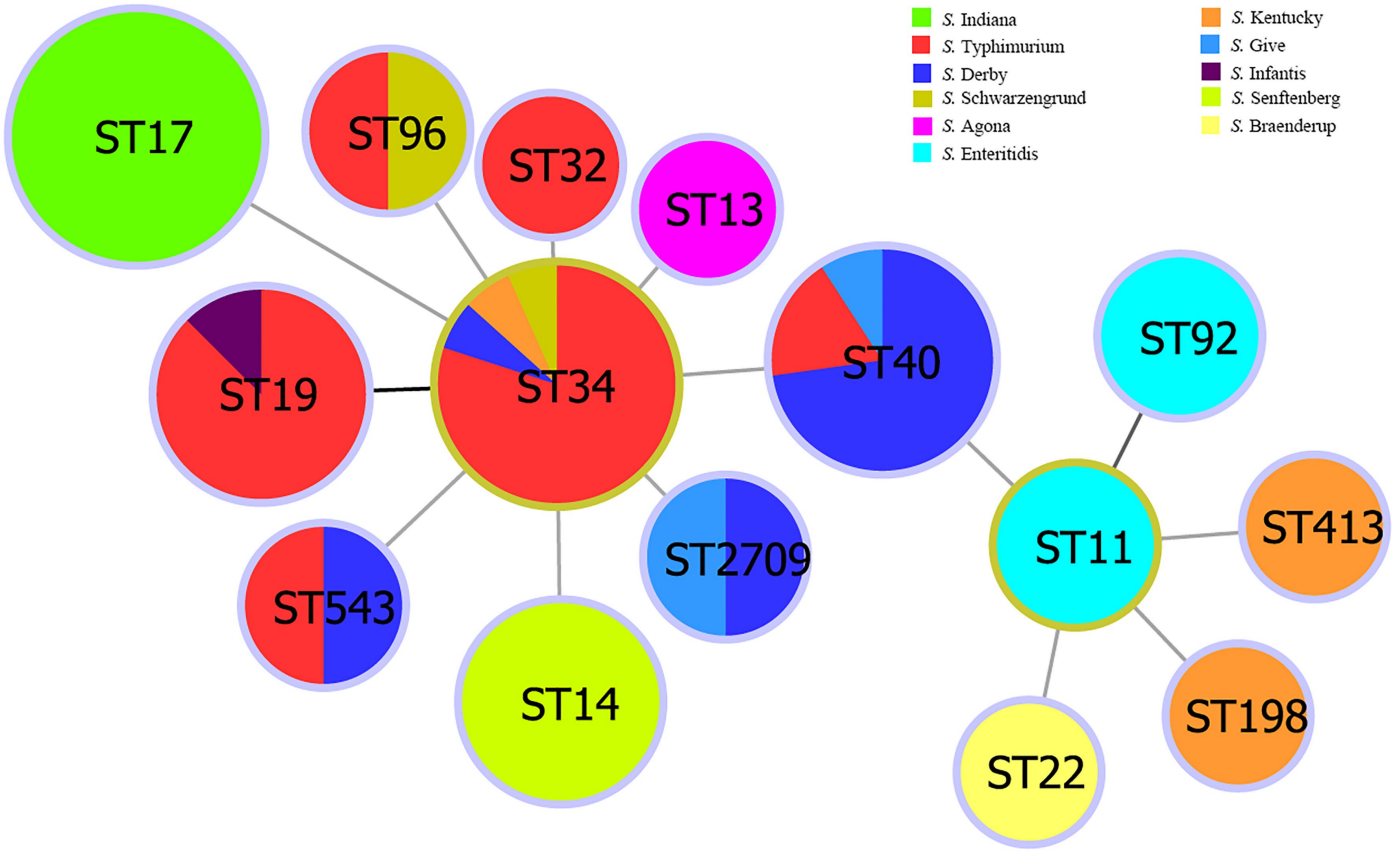
Minimum spanning tree of *Salmonella* isolates from multi-locus sequence typing data.

Multi-locus sequence typing classification showed that the 75 *Salmonella* isolates belonged to 15 ST types (Supplementary Table S1): ST17 (20/75, 26.67%); ST34 (15/75, 20.00%); ST40 (11/75, 14.67%); ST19 (9/75, 12.00%); ST14 (6/75, 8.00%); ST92, ST11, ST96, ST543, and ST2709 (2/75, 2.67%); ST32, ST413, ST22, ST13, and ST198 (1/75, 1.33%). The STs of *S. Indiana* and *S. Senftenberg* were consistent with their serotypes. The *S. Typhimurium* isolates were divided into six STs, the *S. Enteritidis* isolates were divided into two STs, and *S. Kentucky* and *S. Derby* isolates were divided into three STs. The results of the minimum spanning tree-based MLST showed that ST34 was a cloned progenitor and that ST40 evolved from the central ST34 lineage (Figure 2). ST11 isolates were subcloned from the isolate ST40. ST22, ST413, ST198, and ST92 had close genetic relationships, whereas the other STs had close genetic relationships with ST34.

### Analysis of the CRISPR-Cas System in *Salmonella*

**Figure 3:**
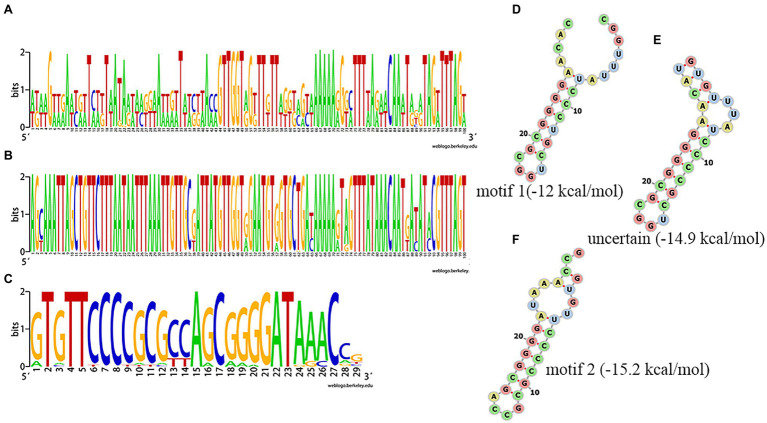
Analysis of clustered regularly interspaced short palindromic repeat (CRISPR) leader and direct sequences (DRs). **(A,B)** Conservation analysis of leader sequences of CRISPR1 and CRISPR2, respectively. **(C)** Conservation analysis of DR sequences. Each logo is consisted of stacks of letters, one stack for each position in the sequence. The height of letters within each stack is measured in bits, with a maximum of two, and reflects the corresponding nucleotide conservation at that position. **(D–F)** Predicted secondary structure and MEF of a partial DR.

Among the 75 *Salmonella* isolates, 73 strains had the same general type I-E structure (mostly CRISPR1-Cas system), comprising CRISPR array, leader sequence and cas genes (*cas2*, *cas1*, *cas6*, *cas5*, *cas7*, *cse2*, *cse1*, and *cas3*). One of the other two had only two orphan CRISPR arrays and the other had no CRISPR-Cas system. The upstream 5' end of the CRISPR array was adjacent to a leader sequence, which could act as a promoter for the pre-crRNA synthesis. Alignment of the leader sequence of approximately 100bp adjacent to the upstream CRISPR array showed that there were large differences in CRISPR1 leaders and that the CRISPR2 leaders were more conserved than the CRISPR1 leaders (Figures 3A, B). The DR sequences of CRISPR1 and CRISPR2 were similar. In the sequence comparison and conservation analysis of DR sequences among all CRISPR arrays (evidence level 3 or 4 in CRISPRCasFinder), 31 types of relatively conserved DR sequences were found, ranging in length from 24 to 29bp, with most being 29bp (Figure 3C and Supplementary Table S1). By analyzing these 31 types of DR sequences using CRISPRmap, we found that five DRs had motifs, including four motifs 1 and one motif 2, and 26 could be classified as existing families, including 21 families 2 and five family 4, suggesting that these DRs were involved in gene expression or DNA metabolism. The results of the MFE secondary structure and MFE prediction showed that a stable stem loop structure was formed by all 31 types of DRs (Figures 3D–F).

In the CRISPR array, the highest numbers of spacers were 33 in CRISPR1 and 34 in CRISPR2, whereas the lowest numbers were seven in CRISPR1 and four in CRISPR2, for a total of 1,607 spacer sequences in CRISPR1 and 1,226 in CRISPR2. After removing duplicates, 517 unique sequences were obtained (274 in CRISPR1 and 243 in CRISPR2), and the length of sequences ranged from 29 to 70bp, with the majority being 32bp. Target prediction was performed on the above 517 spacer sequences using CRISPRTarget. Of these, sixty-one (11.79%) were found to be homologous to bacteriophages or viruses, and 137 (26.49%) were homologous to plasmids. Other spacers targeted bacteria genes of the non-CRISPR array portions of their own genome, which covered all aspects of bacterial life activities, such as genetic information storage and processing, bacterial functional activities, and metabolism. Many genes associated with antimicrobial function, such as multidrug excretion proteins, and penicillin-binding proteins were also found to be homologous.

### CRISPR Typing Analysis

**Figure 4:**
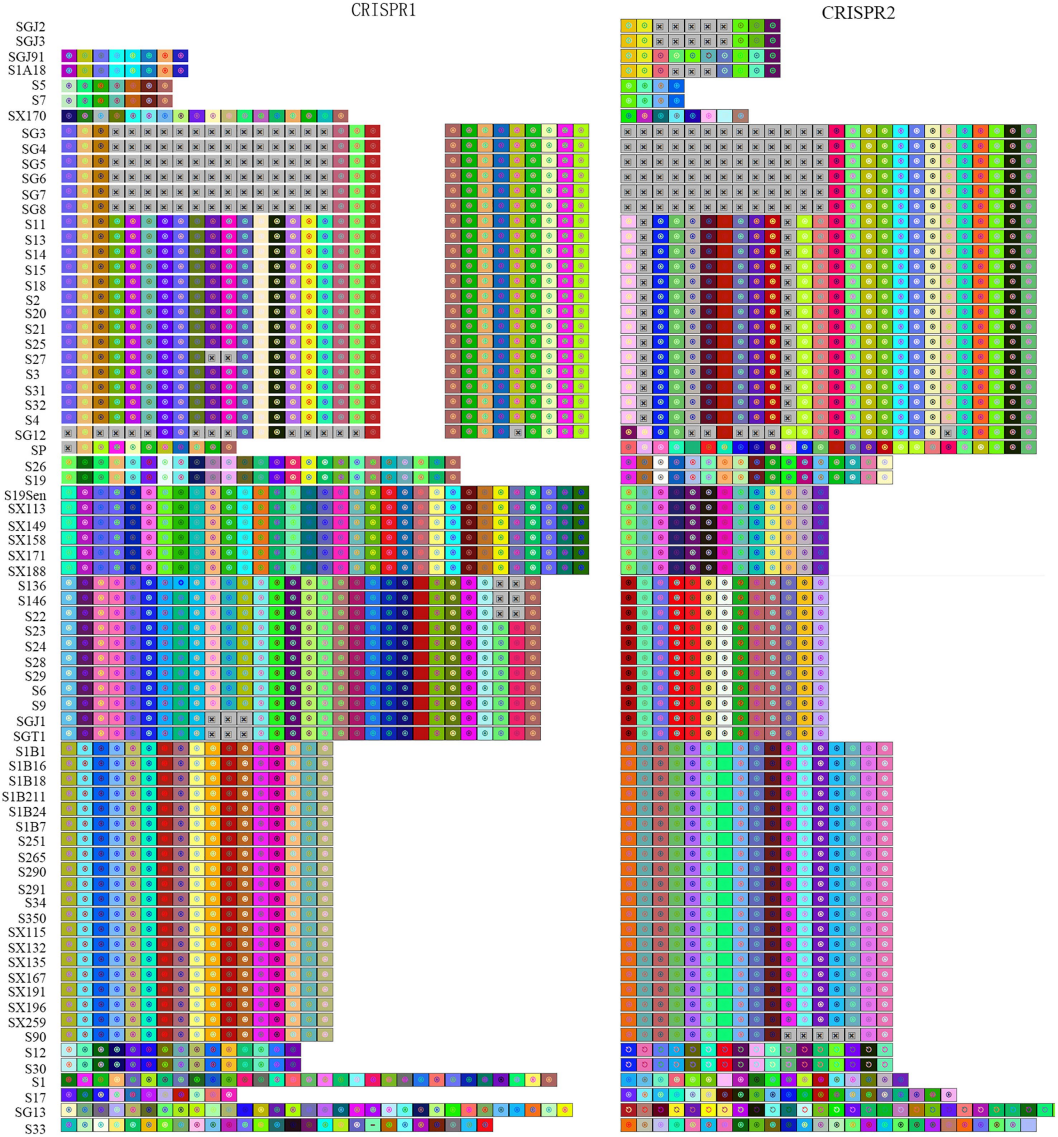
Spacer sequence profiles of CRISPR1 and CRISPR2 in *Salmonella* isolates. Each spacer is represented by a colored square and a geometric symbol based on the CRISPRviz. The gray square with × indicates deleted or missing spacer. The earliest acquired spacer is displayed on the left side and the newly acquired spacer is on the right side.

**Figure 5:**
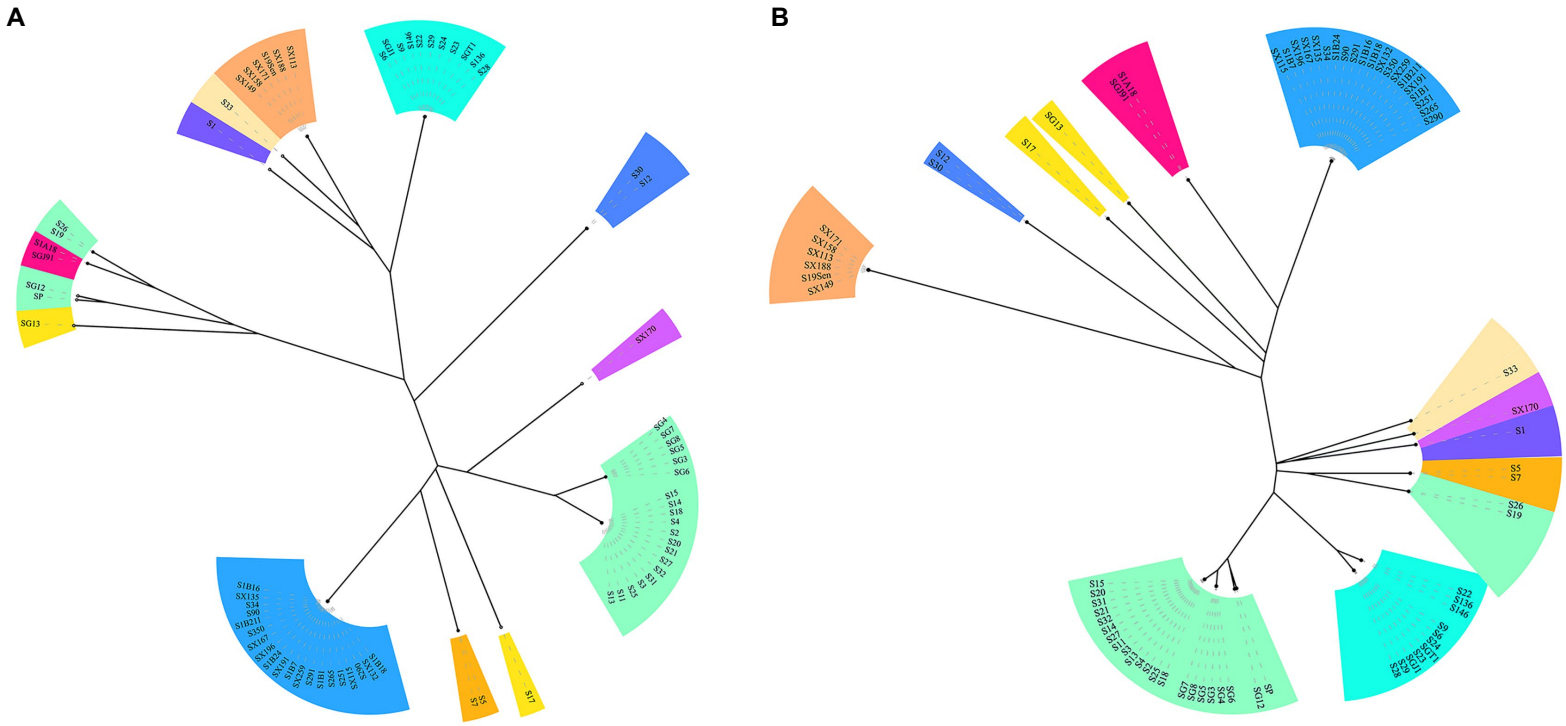
Unweighted pair group method with arithmetic mean (UPGMA) tree of *Salmonella* isolates. **(A)** UPGMA tree based on CRISPR locus spacer pair typing (CLSPT). **(B)** UPGMA tree based on CRISPR locus three spacer sequence typing (CLTSST). The same color indicates the same serotype.

CRISPR typing was performed by combining CRISPR1 and CRISPR2. The resulting profile for each strain after the spacer sequence was arrayed in CCT, as shown in Figure 4, and the strains were found to have high genetic polymorphism. The arrangement of CRISPR spacers of the same serotype was similar or identical. In total, the 72 *Salmonella* strains that had two CRISPR loci were divided into 20 CRISPR types (Figure 4), whereas in CLSPT, 72 *Salmonella* strains were subtyped into 16 different CRISPR types (Figure 5A). Overall, the 72 *Salmonella* strains were subtyped into 17 different CRISPR types in CLTSST (Figure 5B). Among the three typing methods, the values for Simpson’s index of diversity were 87.75, 85.52, and 86.46%, respectively.

To evaluate the relationships between all detected CRISPR types and serotypes, a phylogenetic tree based on the spacer sequences of the CRISPR types was constructed. The results showed that the CRISPR types had a good correspondence with serotypes, whereas strains belonging to the same CRISPR type were usually located on the neighboring branch. For *S. Typhimurium*, four small divergent phylogenetic clusters were observed in CLTSST and CLSPT (Figure 5).

### Association Between CRISPR-Cas System and Antimicrobial Resistance

**Figure 6:**
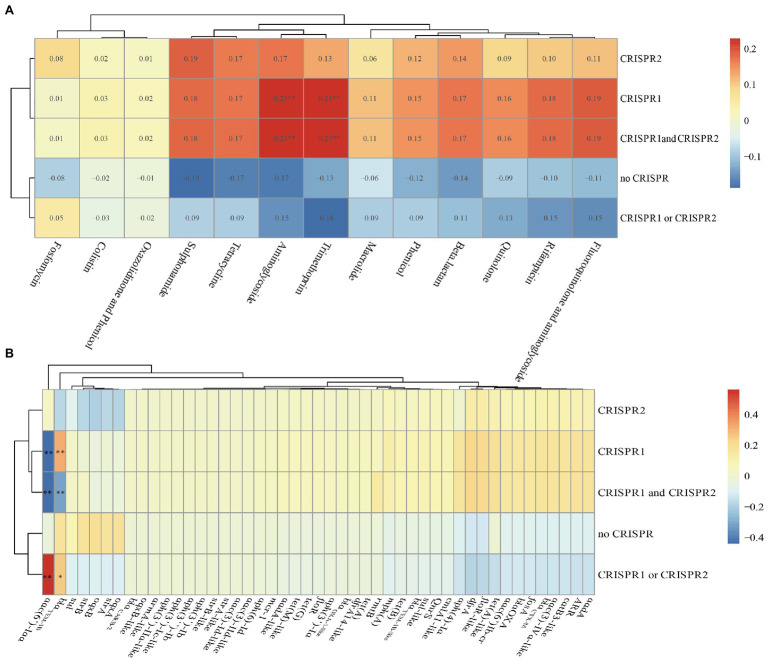
Correlation heat map between CRISPR loci and antibiotics or antibiotic resistance genes in the total of 75 *Salmonella* isolates. **(A)** Correlation between CRISPR loci and antibiotics. **(B)** Correlation between CRISPR loci and antibiotic resistance genes. Correlation is expressed by Spearman correlation coefficient which is indicated by color gradient, red represents positive correlation, and blue represents negative correlation. ^**^ denotes *p*<0.01; ^*^ denotes *p*<0.05. Generally, a correlation coefficient above 0.7 indicates that the relationship is very close; 0.4–0.7 indicates that the relationship is close; and 0.2–0.4 indicates that the relationship is normal.

We found that there were no differences in the numbers of spacers in CRISPR arrays after comparing CRISPR sequences between susceptible and resistant strains (Table 1 and Supplementary Table S1). We also calculated the Spearman’s rank correlation coefficient between the CRISPR and antibiotics (Figure 6A). From the figure, the CRISPR1, CRISPR1, and CRISPR2 were correlation with aminoglycoside and trimethoprim antibiotics with a correlation coefficient of 0.23 (*p*<0.01) which was indicating a modest relationship. Additionally, in total, 47 drug-resistant genes identified in the isolates were analyzed, and we found that the *aac(6')-Iaa* was close positive (the correlation coefficient was 0.561, *p*<0.01) correlation with the CRISPR1 or CRISPR2 and was close negative (the correlation coefficients were −0.441 and −0.435, *p*<0.01) correlation with CRISPR1, CRISPR1, and CRISPR2 (Figure 6B). The *bla*_TEM-1B_ was close positive (*p*<0.01) correlation with the CRISPR1 and was close negative (*p*<0.01) correlation with CRISPR1 and CRISPR2 (Figure 6B). These results indicated that there were no close correlations between CRISPR loci and antibiotics but had close correlations between CRISPR loci and ARGs in *Salmonella* isolates.

## Discussion

Recent reports have shown that the distribution of CRISPR spacer sequences of *Salmonella* is related to the serotype and subtype (Fabre et al., 2012). A CLSPT method based on the newly incorporated spacer for *Salmonella enterica* has been constructed; this is a simplified CRISPR-based typing method with higher consistency compared with traditional serotyping (Li et al., 2014). CRISPR combined with multi-site virulence sequence typing (CRISPR-MVLST) has been established in *Salmonella* and has proven to be a very effective typing technique in some serotypes (Shariat et al., 2013b, 2013a). Two CRISPR spacer sequences of *Salmonella* were combined with two virulence genes (*fimH* and *sseL*) and MLST to type *Salmonella*, and the results showed that the new genotyping method could improve the resolution compared with PFGE (Liu et al., 2011). However, because the above methods ignored differences in other spacer sequences, they showed weaker typing effects. In order to make up for this shortcoming, we developed a new method to distinguish *Salmonella* isolates in this work. Compared with the other subtyping methods (MLST, CCT, and CLSPT), the discrimination of CLTSST (86.46%) was weaker than that of CCT (87.75%), but stronger than those of MLST (85.66%) and CLSPT (85.52%). In these CRISPR-based typing methods, the CCT needed to analyze all spacer sequences in the strain. And the CLTSST only needed to know part of the spacer sequences, which not only had good typing effect, but also greatly reduced the cost. Therefore, the CLTSST could be used as the best method for typing different serotypes. The CLSPT was a simplified version of CCT. It only needed to analyze the newly inserted spacer sequences but ignored the first three spacer sequences that best reflected the original information of the strain and the CLTSST just made up for this deficiency. However, the CLTSST still had some shortcomings. For example, when there was no CRISPR in bacteria, this method could not be used. At the same time, this method still ignored part of the spacer sequence, so this method still needed to be further improved.

In this study, the lengths of different CRISPR arrays varied greatly, with the number of spacers ranging from 4 to 34. In some strains, spacers appeared repeatedly in multiple ways. The repetitive distribution of the spacer might be related to the sequence recombination of the repeat, and the recombination process might be accompanied by an increase and deletion of the spacer, as well as the position change of the spacer. This R-S recombination update could improve the adaptability of the strain. The structural features and sequence functions of *Salmonella* CRISPR were also explored. The DR sequences were different, and part of the spacer sequences was derived from foreign genes. We speculated that under antibiotic selection pressure, the bacterial CRISPR sequence may mutate, allowing the bacteria to acquire exogenous resistant genes and survive. This suggested that the variation in CRISPR DR sequences and the diversity of spacer sequences might be related to the resistance of *Salmonella*. The relationships between the number of CRISPR spacers and antibiotic resistance differed according to the type of antibiotic and the species of the strain. For example, the number of CRISPR spacers was positively correlated with SXT resistance but not correlated with AMP and cefazolin resistance in uropathogenic *E. coli* (Dang et al., 2013). By analyzing the relationships between the CRISPR-Cas system of 263 strains of *E. coli* and drug resistance, researchers found that there were no significant differences in the distributions of cas between sensitive and resistant strains and that drug-resistant plasmids could spread among CRISPR-positive *E. coli* (Touchon et al., 2012). However, the distribution of *Enterococcus faecalis* CRISPR was negatively correlated with bacterial resistance (Palmer and Gilmore, 2010). In this study, there were no close correlations between the CRISPR-Cas system and antibiotics in *Salmonella* isolates. But the *aac(6')-Iaa* was close positive correlation with the CRISPR1 or CRISPR2 and was close negative correlation with CRISPR1, CRISPR1 and CRISPR2. The *bla*_TEM-1B_ was close positive correlation with the CRISPR1 and was close negative correlation with CRISPR1 and CRISPR2. To date, no unified statement of the relationships between bacterial CRISPR and bacterial resistance to antibiotics has been proposed. This may be because of variations related to factors, such as the bacterial species, antibiotic species, host, strain isolation time, and geographic location. Therefore, further studies are needed to assess the relationships between bacterial CRISPR and antibiotic resistance.

## Conclusion

In summary, we identified 517 unique spacers and 31 unique direct repeats in the CRISPR loci of these isolates. Further analysis of the identified spacers and repeats demonstrated specific patterns that harbored certain associations with genotype. We also established the CLTSST method and applied this method to the typing of different serotypes of *Salmonella* to explore the structure and function of CRISPR. Simultaneously, compared with the other three typing methods, we found that the discrimination of CLTSST was weaker than that of CCT, but stronger than those of MLST and CLSPT. In addition, we analyzed the relationships between CRISPR loci and *Salmonella* resistance and found that there were no close correlations between CRISPR loci and antibiotics but had close correlations between CRISPR loci and ARGs in *Salmonella* isolates.

## Data Availability Statement

The datasets presented in this study can be found in online repositories. The names of the repository/repositories and accession number(s) can be found at: https://www.ncbi.nlm.nih.gov/, SAMN19819839, SAMN19819840, SAMN19819841, SAMN19819842, SAMN19819843, SAMN19819844, SAMN19819845, SAMN19819846, SAMN19819847, SAMN19819848, SAMN19819849, SAMN19819850, SAMN19819851, SAMN19819852, SAMN19819853, SAMN19819854, SAMN19819855, SAMN19819856, SAMN19819857, SAMN19819858, SAMN19819859, SAMN19819860, SAMN19819861, SAMN19819862, SAMN19819863, SAMN19819864, SAMN19819865, SAMN19819866, SAMN19819867, SAMN19819868, SAMN19819869, SAMN19820037, SAMN19820038, SAMN19820039, SAMN19820040, SAMN19820041, SAMN19819984, SAMN19819985, SAMN19819986, SAMN19819987, SAMN19819988, SAMN19819989, SAMN19819990, SAMN19819991, SAMN19819992, SAMN19819993, SAMN19819994, SAMN19819995, SAMN19819996, SAMN19819997, SAMN19819998, SAMN19819999, SAMN19820000, SAMN19820001, SAMN19820002, SAMN19820003, SAMN19820004, SAMN19820005, SAMN19820006, SAMN19820007, SAMN19820008, SAMN19820009, SAMN19820010, SAMN19812804, SAMN19812805, SAMN19812806, SAMN19812807, SAMN19812808, SAMN19812809, SAMN19812810, SAMN19812811, SAMN19812510, SAMN19812511, SAMN19812512, SAMN19812513.

## Author Contributions

HW and CuL designed the study and supervised the work. YW, YG, ChL, and BM participated, coordinated, and analyzed the data. CuL wrote the manuscript. All authors approved the final manuscript.

## Funding

This work was supported by the National Natural Science Foundation of China (31830098 and 31772769), the China Agriculture Research System National System for Layer Production Technology (CARS-40-K14), and the Fundamental Research Funds for the Central Universities (SCU2021D006).

## Conflict of Interest

The authors declare that they have no competing financial interests or personal relationships that could have appeared to influence the work reported in this paper.

## Publisher’s Note

All claims expressed in this article are solely those of the authors and do not necessarily represent those of their affiliated organizations, or those of the publisher, the editors and the reviewers. Any product that may be evaluated in this article, or claim that may be made by its manufacturer, is not guaranteed or endorsed by the publisher.

## Footnotes

^1^ https://cge.cbs.dtu.dk/services/ResFinder/

^2^ https://crisprcas.i2bc.paris-saclay.fr/CrisprCasFinder/Index

^3^ http://weblogo.berkeley.edu/logo.cgi

^4^ http://rna.tbi.univie.ac.at/cgi-bin/RNAfold.cgi

^5^ http://bioanalysis.otago.ac.nz/CRISPRTarget/crispr_analysis.html

^6^ https://cge.cbs.dtu.dk/services/MLST/
